# Litter Decomposition of *Imperata cylindrica* in a Copper Tailing Areas With Different Restoration History: Fungal Community Dynamics and Driving Factors

**DOI:** 10.3389/fmicb.2021.780015

**Published:** 2021-11-22

**Authors:** Tong Jia, Xuerong Wang, Tingyan Guo, Baofeng Chai

**Affiliations:** Shanxi Laboratory for Yellow River, Shanxi Key Laboratory of Ecological Restoration on Loess Plateau, Institute of Loess Plateau, Shanxi University, Taiyuan, China

**Keywords:** fungal community, litter properties, decomposition dynamics, *Imperata cylindrica*, copper mining area

## Abstract

Microorganisms drive litter decomposition while maintaining the chemical cycle of ecosystems. We used the dominant vegetation (*Imperata cylindrica*) in the mining area selected for this study for this experiment to explore fungal community characteristics, key fungal groups, and their associative driving factors during *I. cylindrica* litter decomposition. Maximum litter C/N values occurred 100days after the commencement of the decomposition experiment during all different recovery years in this copper tailings area. Heavy metals in litter [copper (Cu), zinc (Zn), plumbum (Pb), and cadmium (Cd)] accumulated gradually with decomposition. The dominant fungal phyla observed in the community were Ascomycota and Basidiomycota, while the classes Sordariomycetes and Eurotiomycetes significantly increased as litter decomposition progressed. Degrees of connectivity and interaction between fungal communities were highest during the early litter decomposition stage. Sordariomycetes, Dothideomycetes, and Leotiomycetes all played critical roles in maintaining fungal community relationships. The effect of physicochemical properties and enzyme activities in *I. cylindrica* litter was significant on the dominant fungi, while driving factors that affected fungal communities differed over different recovery stages. Total nitrogen (TN), heavy metals, pH, and enzyme activities in the little were significantly correlated with fungal community composition. Litter properties throughout the litter decomposition process mainly affected the dynamics of the fungal community structure. The main environmental factors that affected fungal community structure were copper content and pH. *Dichotomopilus*, *Trichoderma*, *Knufia*, *Phialophora*, *Oxyporus*, and *Monocillium*, which all played important roles in litter decomposition, positively correlated with heavy metals, sucrase, and catalase. Finally, results from this study will help us better clarify litter decomposition mechanisms in degraded ecosystems as well as provide a scientific basis for improving species cycling and nutrient transformation efficiency in mining ecosystems.

## Introduction

Although mineral resources are the basis of national economic planning, mining production activities over long periods will have a severe impact on the ecological environment of mining areas. Furthermore, mining activities destabilize the integrity of mountain structures and vegetation coverage while destroying existing landforms (Huang, 2020). The construction of mining areas also displaces enormous amounts of soil while also disrupting the daily lives of residents (Wang, 2020). Additionally, vast amounts of wastewater and solid waste material are produced during mineral resource processing, which has had a catastrophic effect on local water resources and soil ecosystems (Yan and Feng, 2010). In other words, soil organic matter and the nutrient quality of mining areas are extremely poor.

Phytoremediation is a popular method used to alleviate soil organic matter and soil nutrient content degradation in mining areas. It is used to improve the carbon sequestration capacity of aerial components of plants, the root secretion capacity of plants, and the microbial decomposition capacity of plant litter in mining areas (Meng et al., 2018). Plant litter accumulates year by year with vegetation restoration in mining areas. Litter decomposition is a critical factor in ecosystem nutrient transformation processes. Litter properties and microbial community characteristics are also important factors that determined litter decomposition rates. Additionally, environmental factors have important effects on litter decomposition processes. Accordingly, this study explored the biological and abiotic factors that take place during the litter decomposition process in a Chinese copper tailings mining area to help improve species cycling and nutrient conversion efficiency. This study also offers an ecological restoration approach that can be used in copper tailings areas under severe heavy metal pollution.

Saprophytic fungi are widely known to be the main litter decomposers, which secrete extracellular enzymes that decompose cellulose, lignin, and other macromolecular compounds. Fungi are known to have effective and efficient enzymatic systems, some of which can produce extensive hypha. Fungal hyphae penetrate litter where they subsequently decompose carbohydrates, pectin, lignin, and cellulose. At the same time, enzymes in litter can help alter the structure and chemical composition of litter while decomposing complex organic matter into soluble amino acids or other small molecular compounds (Pfeiffer et al., 2013). Most fungi that belong to the Ascomycota phylum can secrete cellulase and hemicellulase, which both play critical roles in litter decomposition (Snajdr et al., 2011). Additionally, Basidiomycetes species are the main decomposers of lignin, and their relative abundance has been shown to peak during the latter stage of litter decomposition (Wang et al., 2019a,b). Moreover, it is known that fungal community succession takes place during litter decomposition. *Aureobasidium*, *Mucor*, and *Aspergillus* are the dominant fungal genera during the early litter decomposition stage, being able to utilize simple sugars and starch. However, over time these fungi genera are gradually replaced by cellulose-decomposing and lignin-decomposing fungi, such as *Trichoderma*, *Marasmius*, and *Mycena*, all of which can secrete cellulase, peroxidase, and polyphenol oxidase. These fungi also play important roles in cellulose and lignin degradation during litter decomposition (Yan, 2011). *Mortierella* and *Penicillium* are known to dominate during the latter litter decomposition stage (Zhang et al., 2003). However, although many relevant studies have focused on natural ecosystems, little is known about litter decomposition characteristics within degraded environments, such as mining areas.

The Northern Copper Mine, Shanxi Province, is China’s largest mine not based on coal, which producing 7 million tons of copper tailings annually (Wang et al., 2018). Through soil analysis, a previous study had determined that the soil of this copper tailings area has been contaminated with various heavy metal elements, the most important being arsenic (As), cadmium (Cd), copper (Cu), plumbum (Pb), chromium (Cr), and zinc (Zn; Jia et al., 2019). Dominant plant species include *I. cylindrica*, *Bothriochloa ischaemum*, and *Artemisia sacrorum* to name a few, all of which yield enormous amounts of litter at the end of the growing season. Based on this, we conducted a 460day *in situ* litter decomposition experiment. As the study object, *I. cylindrical* litter was selected to explore the dynamics of fungal community structure and composition during different decomposition stages using high-throughput sequencing. We also measured physicochemical properties and extracellular enzyme activities in litter. The objective of this study was to answer the following three questions: (1) How did decomposition rates and nutrient content change during litter decomposition in this copper tailings area? (2) In what way did fungal community characteristics and their interactive relationships dynamically change? (3) What were the driven factors of fungal community succession in litter during the litter decomposition process?

## Materials and Methods

### Site Description and Litter Sampling

Initial construction on the Shibahe tailings dam (lat 35°15′~35°17′ N, long 118°38′~111°39′ E) began in 1969. This copper tailings dam is a branch of the expansive Northern Copper Mine in China’s Shanxi Province. The Shibahe tailings dam is currently subdivided into 16 smaller sub-dams. The region is under the influence of a continental monsoon climate, where the average annual temperature is 13.5°C and annual precipitation is 631mm (Jia et al., 2019). Our experiments were conducted at three of the 16 three sub-dams: the S516 sub-dam, the S536 sub-dam, and the S560 sub-dam, whose representative phytoremediation years are 50, 22, and 5 (Supplementary Table S1; Jia et al., 2019).

In April 2019, we collected a total of nine *I. cylindrica* litter samples from each of the three sub-dams. Litter samples were then divided into two, where one was used as initial litter (D0) for high-throughput sequencing and the other was naturally air-dried to use in litter decomposition bags. The size of the nylon mesh bags was 20cm×20cm, with an aperture of 1mm×1mm. A total of 8g of *I. cylindrica* litter was placed into each nylon mesh bag. In May 2019, three sample plots were established in each sub-dam for which three mesh bags were added to each plot. This provided a total of 27 litter bags (Supplementary Figure S1). Litter samples were collected after being allowed to decompose for 100days (D100), 200days (D200), and 460days (D460). This provided a total of 36 litter samples. These littler samples were subsequently divided into two. One was stored (−20°C) for high-throughput sequencing, and the other was stored (4°C) to ascertain physiochemical properties and enzyme activities.

### Mass Litter Residual Rate and Chemical Properties

Litter bags were collected, and sediment removed, after which the fresh weight of litter (Wi) was measured. A portion of the samples was dried at 65°C to a constant weight, and water content (P) was measured. The mass residual rate of litter was then calculated (Zhang et al., 2019). The mass residual rate (%)=W_i_^*^(1-P)/W_0_. Here, *W_0_* denotes the dry weight of the initial litter bag.

An elemental analyzer (vario EL/MACRO cube, Elementar Analysensysteme GmbH, Hanau, Germany) was used to measure total carbon (TC) and total nitrogen (TN) content in litter samples. Before measuring litter pH, litter water (1:20 mass/volume) suspensions were shaken for 30min (Fioretto et al., 2000). Heavy metal (Cu, Zn, Pb, and Cd) concentrations in the litter were measured using atomic absorption spectrometry (Agilent Technologies 200 Series AA, United States). Additionally, 3,5-Dinitrosalicylic acid colorimetry was used to measure litter sucrose and cellulase content; phenol-sodium hypochlorite colorimetry was used to measure urease content; potassium permanganate titration was used to measure catalase content; and the disodium phenyl phosphate colorimetric method was used to measure phosphatase content (Jia et al., 2019).

### DNA Extraction, PCR Amplification, and Miseq Sequencing

Before being filtered through sterile membranes (0.2μm pore size; Millipore, Jinteng, Tianjin, China), we washed litter samples three separate times in a sterile phosphate buffer solution (PBS: NaCl, KCl, Na_2_HPO_4_, and KH_2_PO_4_). Filtered samples were then sealed in sterile centrifuge tubes before extracting microbial DNA. Following the manufacturer’s instructions, the E.Z.N.A.^®^ Soil DNA Kit (Omega Bio-Tek, Norcross, GA, United States) was used to extract microbial DNA in litter samples. Additionally, we used the NanoDrop ND-1000 UV–Vis Spectrophotometer (NanoDrop Technologies, Wilmington, DE, United States) for DNA quantification. Primers ITS1F (5′-CTTGGTCATTTAGAGGAAGTAA-3′) and ITS2 (5′-GCTGCGTTCTTCATCGATGC-3′) were used as the internal transcribed spacer (ITS) gene copy numbers for all samples. Sequencing was done at Shanghai Majorbio Bio-pharm Technology (Shanghai, China), using the MiSeq platform (Illumina, Inc., United States). We submitted raw sequencing data to the National Center for Biotechnology Information Sequence Read Archive^1^ under the project accession number PRJNA764552.

### Statistical Analysis

QIIME was used to integrate the original FASTQ format data (Caporaso et al., 2010). USEARCH (ver. 7.^2^) was used for the verification and removal of chimeric sequences. The operational taxonomic unit (OTU) partition threshold was established at a 97% sequence similarity of the classification results, after which it was used for fungal community diversity and relative abundance calculations. To procure species classification data that correspond to each OTU, the RDP Classifier^3^ was employed for the classification and analysis of each OTU sequence. Using the UNITE8.0 fungi database, the reliability threshold was determined to be 70%.

SPSS ver. 24.0 was used to analyze the physicochemical properties of litter during different decomposition stages, while Duncan’s multiple range test was used in one-way ANOVA. A Student’s *t* test was used to compare both fungal diversity and richness during the different decomposition stages. Moreover, we applied non-metric multidimensional scaling (nMDS) analysis to ascertain the fungal community structure based on Bray–Curtis dissimilarity, while we used analysis of similarities (ANOSIM) to ascertain intergroup differences. Additionally, we applied variance inflation factor analysis to eradicate high multicollinearity in environmental factors employing the “vegan package” in R3.5.3. We further employed Canoco 5.0 (Microcomputer Power, United States) for redundancy analysis. Finally, Gephi (an interactive platform) was used to analyze and envisage networks. Co-correlation network properties, such as the average degree, the network density, the average clustering coefficient, the network diameter, and the average path length, were calculated (Jiao et al., 2020). The higher that the average degree, the average clustering coefficient, and the network density were, the closer the network connection was assumed to be. Additionally, a lower path length and network diameter were indicative of a closed network connection (Ma et al., 2016).

## Results

### Litter Properties Throughout the Decomposition Process

The decomposition rate of *I. cylindrica* litter was higher during the early decomposition stage but then decreased after 100days of decomposition (Supplementary Figure S2). During the latter decomposition stage, the range of the remaining litter mass was from 27.05 to 67.14%, and the mass residual rate of litter in the S536 sub-dam was significantly higher compared to the S560 and S516 sub-dams (*p*<0.05).

The nutrient content of litter decreased as litter decomposition progressed (*p*<0.05). The C/N ratio reached a peak after 100days of decomposition and then significantly decreased. During the early decomposition stage, the litter C/N ratio in the S536 sub-dam was significantly higher compared to the other sub-dams. Heavy metal (Cu, Zn, Pb, and Cd) content gradually accumulated as decomposition progressed. and except for Zn, these heavy metals significantly increased throughout the decomposition process (*p*<0.05). Moreover, litter pH increased throughout the decomposition process in the S516 sub-dam (*p*<0.05), reaching a maximum after 200days of decomposition in the S536 and S560 sub-dams (Supplementary Table S2).

No consistent change was observed in *I. cylindrica* litter enzyme activities throughout the entire decomposition process in all sub-dams (Supplementary Table S3). However, catalase activity in litter significantly exceeded the initial decomposition stage (D0) in the S536 and S560 sub-dams. Litter cellulase activity in these two sub-dams was significantly higher at D100 than the other decomposition stages, while urease activity reached a maximum at D200 (Supplementary Table S3). Sucrase activity in litter significantly increased throughout the decomposition process in the S536 sub-dam, while it first increased before decreasing again throughout the decomposition process in the S516 and S560 sub-dams (*p*<0.05). Compared to the other sub-dams, sucrase activity in the S560 sub-dam was significantly higher (*p*<0.05) after 200days of decomposition (D200).

### Taxonomic Distribution and Fungal Diversity

**Figure 1:**
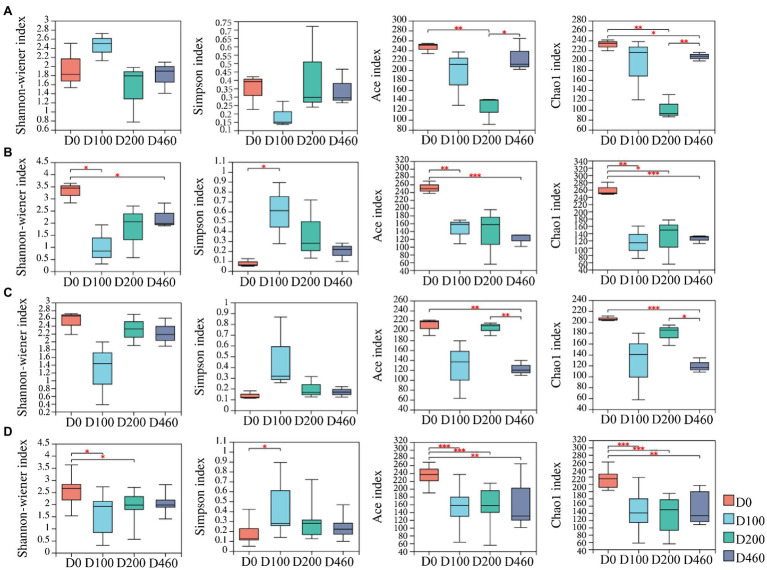
The dynamics of litter fungal community diversity indexes in S516 **(A)**, S536 **(B)**, S560 **(C)**, and the whole copper mining area **(D)**. Significance levels were denoted with ^**^p<0.01 and ^***^p<0.001.

A total of 993 fungal OTUs were obtained from litter samples based on 97% sequence similarity. Specie richness (i.e., the ACE and Chao1 indices) of the litter fungi community decreased at D200 before increasing again in the S516 sub-dam (*p*<0.05). The Shannon, ACE, and Chao1 indices decreased after 100days of decomposition (D100) in the S536 sub-dam (*p*<0.05). However, the richness index of the fungi community in litter decreased at the D460 stage in the S560 dam (*p*<0.05; Figure 1).

**Figure 2:**
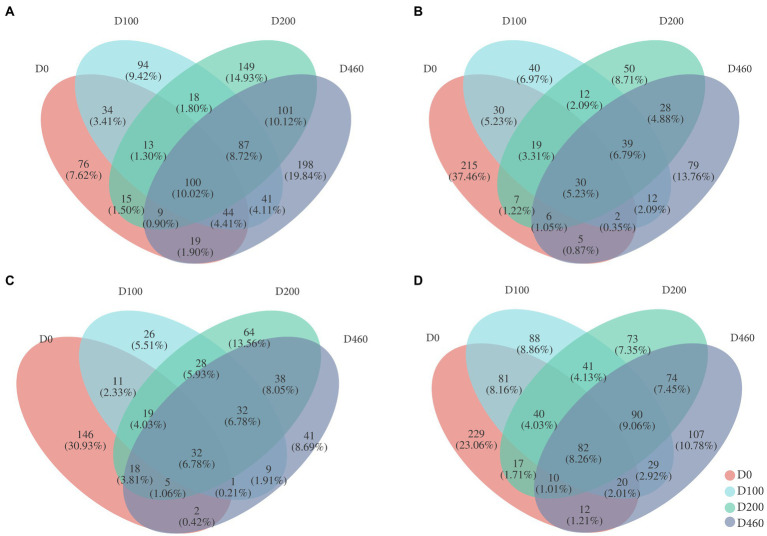
Venn diagrams of litter fungi at OTU level in S516 **(A)**, S536 **(B)**, S560 **(C)**, and the whole copper mining area **(D)**. The percentage written in parentheses means the special species as a percentage of total OTUs.

Results from this study showed that common OTU numbers in each sub-dam throughout the different decomposition stages were 100 (S516), 30 (S536), and 32 (S560), accounting for 10.02, 5.23, and 6.78% of the total OTUs, indicating compositional differences in fungi communities during the different decomposition stages. We found 215 and 146 special OTUs in the litter, accounting for 37.46 and 30.93% of total OUTs at the D0 stage in the S536 and S560 sub-dams, respectively, and 76 special OTUs in the litter were found at the D0 stage in the S516 sub-dam. This was indicative of the significant differences between litter fungi communities and litter decomposition processes (Figure 2).

### Fungal Community Composition Among the Different Litter Decomposition Stages

**Figure 3:**
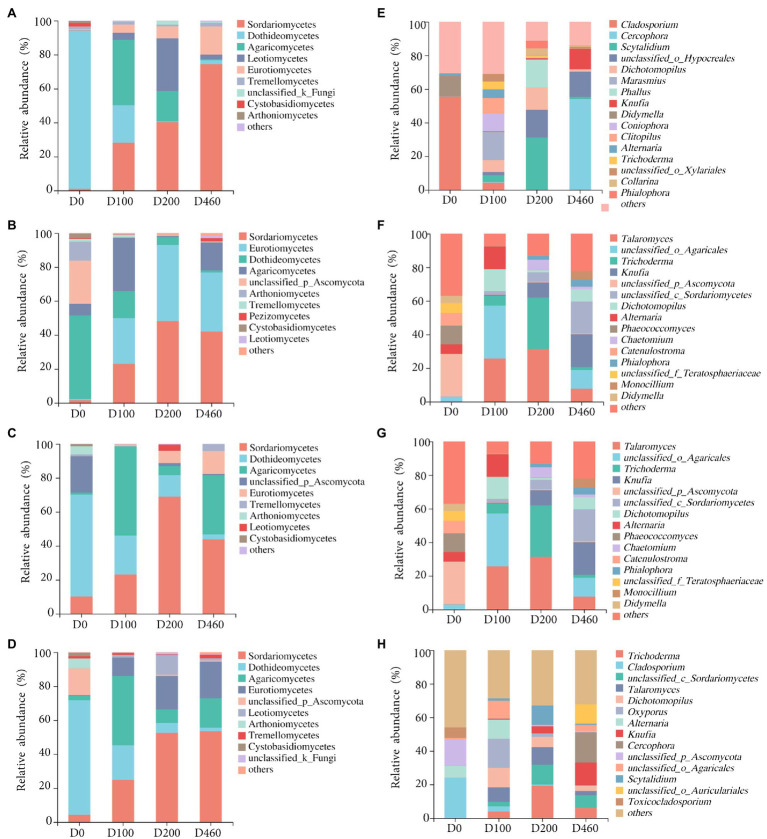
Litter fungal community compositions with relative abundance greater than 1 and 4% at class **(A-D)** and genus level **(E-H)**, respectively, in S516 **(A**,**E)**, S536 **(B**,**F)**, S560 **(C**,**G)**, and the whole copper mining area **(D**,**H)**.

**Figure 4:**
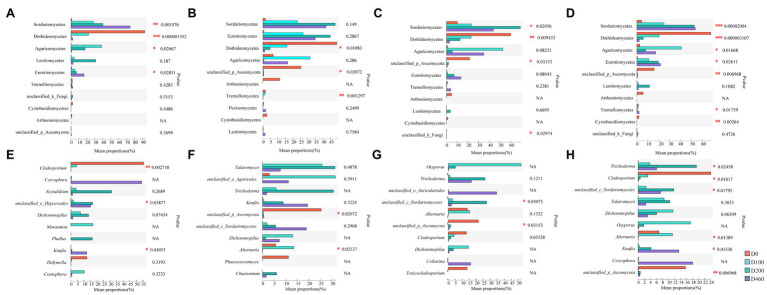
Difference of dominant fungi classes **(A-D)** and genera **(E-H)** at four litter decomposition stages in S516 **(A**,**E)**, S536 **(B**,**F)**, S560 **(C**,**G)**, and the whole copper mining area **(D**,**H)**. Significance levels were denoted with ^**^p<0.01 and ^***^p<0.001.

Ascomycota and Basidiomycota were the dominant fungi in *I. cylindrica* litter, with a 99.41% relative fungal community abundance. Ascomycota was the dominant fungi during the initial litter decomposition stage (D0), with an 88.68% relative abundance. At a class level, Dothideomycetes was the dominant fungal group at the D0 stage, with a 49.19% relative abundance (Figures 3, 4). Dothideomycetes abundance decreased as litter decomposition progressed (*p*<0.05; Figures 3, 4). On the other hand, Sordariomycetes significantly increased as litter decomposition progressed (*p*<0.05). Similarly, Eurotiomycetes also increased with litter decomposition and was affected by litter decomposition in the S516 sub-dam (*p*<0.05; Figures 3, 4). Overall, the relative abundance of Agaricomycetes peaked at D100 (Figure 3).

We observed significant differences in the composition of fungal communities in the litter at a genus level. In the S516 sub-dam, *Cladosporium* was the dominant fungal genus during the initial litter decomposition stage (D0), and it decreased as litter decomposition progressed (*p*<0.05; Figure 4E). However, *Cercophora*, *Scytalidium*, *Dichotomopilus*, and *Knufia* all increased as litter decomposition progressed. The dominant fungi genera were *Talaromyces* and *Trichoderma* during the intermediate and later stages of litter decomposition, respectively (Figure 3). Overall, we observed significant differences among *Trichoderma*, *Cladosporium*, *Alternaria*, and *Knufia* throughout the different decomposition stages (*p*<0.05; Figure 4H).

### Correlations Among Different Fungal Communities

**Figure 5:**
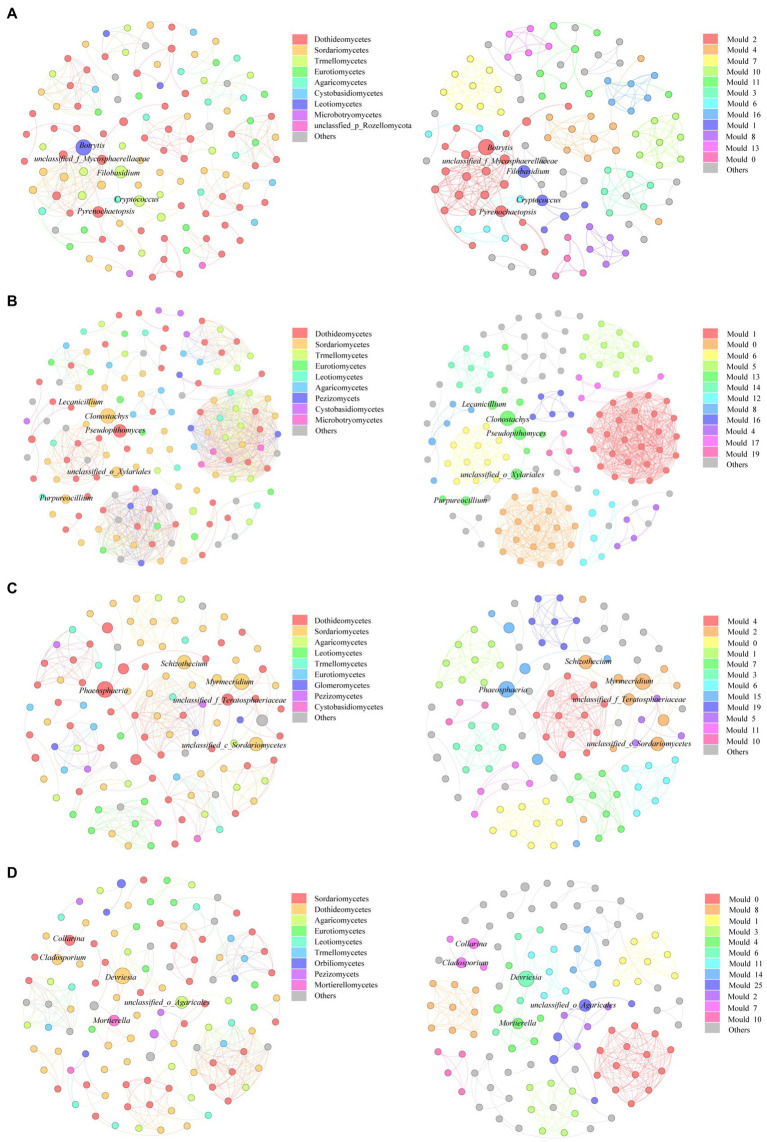
Change of fungal community co-correlation network at D0 **(A)**, D100 **(B)**, D200 **(C)**, and D460 **(D)**. Each node represented a fungal genus, and the line represented a significant correlation between two genera (*p*<0.001). The nodes in the left figure of each decomposition stage were colored by class level, and the nodes in the right figure were colored by module. The top 10 litter fungal classes and modules were defined, and the other microbial were defined as others.

We constructed a genus level-based fungal community co-correlation network to explore changes in fungal community relationships during the litter decomposition process (Figure 5). Results showed that the fungal community network contained 120 nodes and 270 edges during the initial decomposition stage (D0). The number of nodes and edges increased after 100days of decomposition (D100) but decreased after 200days of decomposition (D200). Throughout the different decomposition stages, the proportions of positive fungal community correlations reached 100%, indicating that fungal communities coexisted during the litter decomposition process. Results showed that the average degree, the network density, and the average clustering coefficient of the fungal network were all highest at the D100 stage, indicating that degrees of connectivity and interaction among fungal communities were highest during the early litter decomposition stage. Additionally, to a certain extent, the degree of modularization reflected the degree of community functional diversity. The degree of fungal network modularization during the different decomposition stages was relatively high, which indicated that the functional diversity of the fungal community was high during the litter decomposition process (Table 1). The key fungal groups that maintained fungal network connectivity also changed during the litter decomposition process. *Botrytis* had the highest betweenness centrality (BC) value at the D0 stage. *Cladostachy*s was gradually replaced by *Clonostachys* and *Phaeosphaeri*a as litter decomposition progressed. The role that *Devriesia* played was important in maintaining relationships among fungal communities at the D460 stage (Table 2).

**Table 1:** Properties of litter fungal community correlation network as decomposition progressed.

Topological properties	D0	D100	D200	D460
Nodes	120	148	112	114
Edges	270	761	300	265
Average degree	4.500	10.284	5.357	4.649
Network density	0.038	0.07	0.048	0.041
Modularity	0.867	0.708	0.871	0.84
Average clustering coefficient	0.87	0.94	0.912	0.93
Average path length	2.261	1.124	1.21	1.067
Positive correlation	100%	100%	100%	100%

**Table 2:** Key fungal genera in correlation network during the litter decomposition process.

Decomposition stage	Genus	Class	BC
D0	*Botrytis*	Leotiomycetes	143.0
	*Filobasidium*	Tremellomycetes	88.0
	*Pyrenochaetopsis*	Dothideomycetes	71.0
	*Cryptococcus_f_Tremellaceae*	Tremellomycetes	69.0
	*unclassified_f_Mycosphaerellaceae*	Dothideomycetes	48.0
D100	*Clonostachys*	Sordariomycetes	29.0
	*Pseudopithomyces*	Dothideomycetes	23.5
	*unclassified_o_Xylariales*	Sordariomycetes	16.0
	*Lecanicillium*	Sordariomycetes	9.0
	*Purpureocillium*	Sordariomycetes	9.0
D200	*Phaeosphaeria*	Dothideomycetes	12.0
	*Myrmecridium*	Sordariomycetes	11.7
	*unclassified_c_Sordariomycetes*	Sordariomycetes	9.0
	*Schizothecium*	Sordariomycetes	9.0
	*unclassified_f_Teratosphaeriaceae*	Dothideomycetes	6.3
D460	*Devriesia*	Dothideomycetes	5.0
	*unclassified_o_Agaricales*	Agaricomycetes	3.0
	*Mortierella*	Mortierellomycetes	2.3
	*Cladosporium*	Dothideomycetes	2.0
	*Collarina*	Sordariomycetes	2.0

### Fungal Community Structure and Environmental Variable Correlations

**Figure 6:**
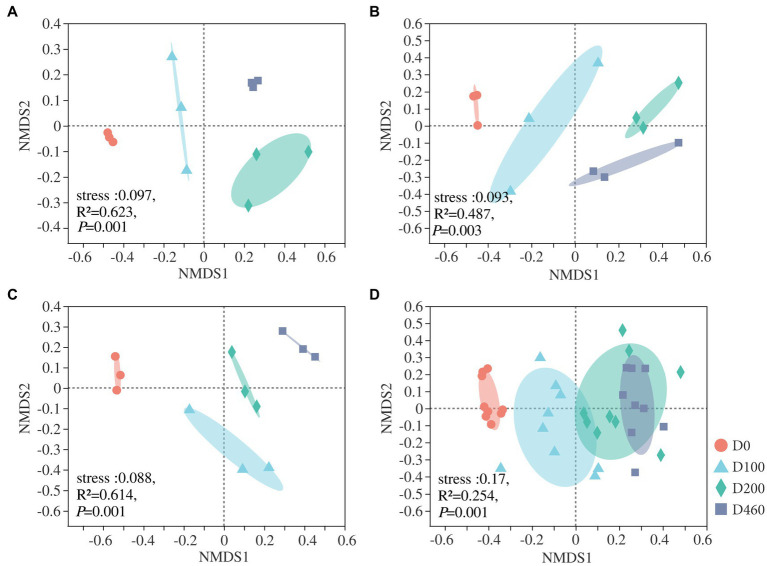
The nMDS of litter fungal community at four decomposition stages in S516 **(A)**, S536 **(B)**, S560 **(C)**, and the whole copper mining area **(D)**.

**Figure 7:**
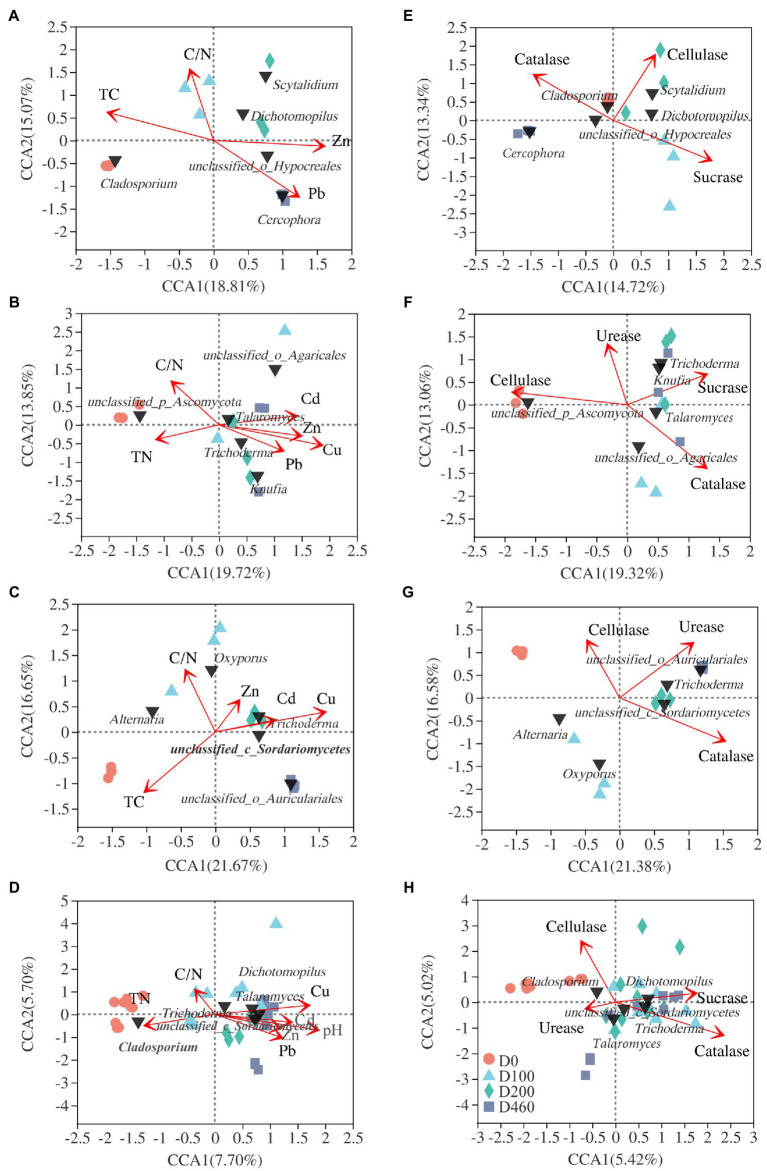
CCA analysis of the top 5 fungal genera (black triangle) and litter properties **(A-D)** and enzyme activities **(E-H)** and fungal community structure in S516 **(A**,**E)**; S536 **(B**,**F)**; S560 **(C**,**G)**; and the whole copper mining area **(D**,**H)**.

At an OUT level, nMDS analysis was conducted on fungal communities at all decomposition stages. Results showed that *I. cylindrica* litter decomposition significantly affected fungal community structure during the different recovery stages (S516: *R*^2^=0.623, *p*=0.001; S536: *R*^2^=487, *p*=0.003; S560: *R*^2^=0.614, *p*=0.001; Figure 6). Canonical correlation analysis results showed that physicochemical properties and enzyme activities in litter significantly affected the dominant fungi in litter (*p*<0.05), while driving factors affecting fungal communities in litter also varied during the different recovery stages (Figure 7). In the S516 sub-dam, driving factors that caused changes to the fungal community structure were Zn (*R*^2^=0.776, *p*=0.003), C/N (*R*^2^=0.740, *p*=0.006), TC (*R*^2^=0.734, *p*=0.004), and Pb (*R*^2^=0.662, *p*=0.016). Moreover, the key factors that affected fungal community structure in the S536 sub-dam were Cu (*R*^2^=0.731, *p*=0.001) and Zn (*R*^2^=0.579, *p*=0.016). Driving factors that significantly affected the fungal community structure in the S560 sub-dam were Cu (*R*^2^=0.896, *p*=0.002), TC (*R*^2^=0.667, *p*=0.01), and C/N (*R*^2^=0500, *p*=0.041). Overall, TN, heavy metal content (Cu, Zn, Pb, and Cd), pH, and enzyme activities (sucrose and catalase) significantly correlated with fungal community composition (*p*<0.05). Variance partitioning analysis results showed that litter properties and extracellular enzyme activities accounted for 13.15 and 7.10% of fungal community structure, respectively. Litter properties during the litter decomposition process primarily affected changes in fungal community structure, while the Cu content (*R*^2^=0.877, *p*=0.002) and pH level (*R*^2^=0.866, *p*=0.002) were the main ecological factors that affected fungal community structure (Supplementary Figure S3).

**Figure 8:**
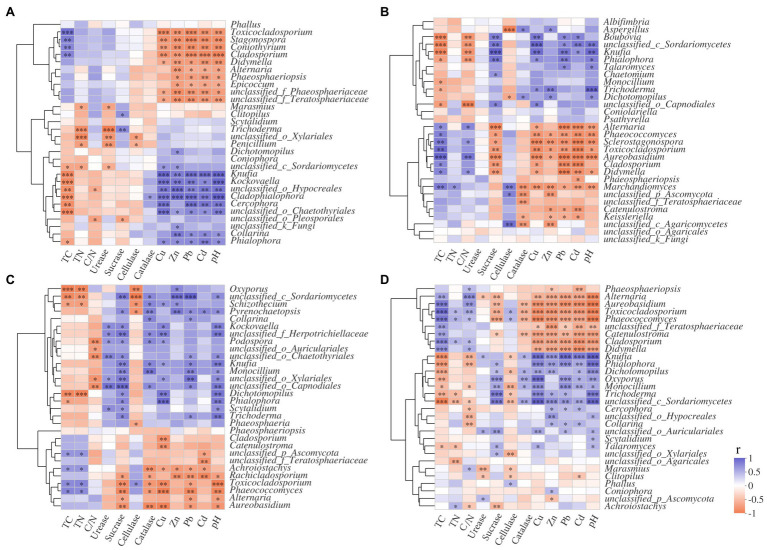
Correlation analysis of litter properties and dominant fungi genera in S516 **(A)**, S536 **(B)**, S560 **(C)**, and the whole copper mining area **(D)**. Different colors denoted the relative coefficients. Significance levels were denoted with ^**^p<0.01 and ^***^p<0.001.

In this study, *Trichoderma* and *Clitopilus* positively correlated with sucrase in the S516 sub-dam (Figure 8A), which can potentially promote cellulose decomposition. *Boubovia*, *Knufia*, and *Phialophora* positively correlated with sucrase, and *Marchandiomyces* significantly correlated with cellulase in the S536 sub-dam (*p*<0.05; Figure 8B), indicating the important roles that these fungi groups may play in maintaining ecosystem carbon cycling. Generally, *Dichotomopilus*, *Trichoderma*, *Knufia*, *Phialophora*, *Oxyporus*, and *Monocillium* significantly and positively correlated with heavy metal content (*p*<0.05), indicating that these genera possess a certain heavy metal tolerance. Moreover, these fungal genera significantly and positively correlated with sucrase and catalase content (*p*<0.05), indicating the critical role that they play in litter decomposition within this copper tailings area (Figures 8C,D).

## Discussion

### Characteristics of Litter Decomposition and Nutrient Release During Different Recovery Stages

Litter decomposition rates closely correlate with their corresponding chemical properties, while TC, TN, total phosphorus (TP), and ecological stoichiometry in a litter affect litter decomposition rates (Sun et al., 2019). It has been reported that litter decomposition rates positively correlate with the initial TN and TP content in the litter but negatively correlate with total organic carbon, lignin, and cellulose content (Cao et al., 2016). In this study, the initial TN content in *I. cylindrica* litter in the S516 and S560 sub-dams is significantly higher compared to the S536 sub-dam, providing more N for microbial growth. This could potentially be the reason that litter decomposition rates in the S516 and S560 sub-dams were higher compared to the S536 sub-dam. Results show that litter decomposition strongly affected Cu, Zn, Pb, and Cd accumulation, which is consistent with a previous study (Sheng, 2009). It has been reported that multivalent metal ions can form highly stable complexes with humus during litter decomposition (Yue et al., 2019). Therefore, more metal ions bind to humus as litter decomposition progress. This indicates that litter may act as an effective metal storage pool; namely, litter absorbs metals from contaminated soil, which may be highly significant in the remediation of heavy metal contaminated soil.

### Fungal Community Succession During Litter Decomposition

Results show that fungal community diversity and richness significantly decreased as litter decomposition progressed. It has been reported the role that fungal communities play primarily occurs during the early litter decomposition stage (Baldrian, 2017). Thus, fungal richness gradually decreases as litter decomposition progresses. Dominant litter microbes are the primary decomposers in ecosystem material cycling processes, which determines litter decomposition rates (Gavito et al., 2021). Ascomycota is the dominant fungal phylum during the early litter decomposition stage, a phylum that is considered an early fungal decomposer (Zhang et al., 2017). Moreover, in this study Ascomycota is the dominant fungal phylum in all samples and is gradually replaced by Basidiomycota as litter decomposition progressed, which consistent with results from a previous study (Purahong et al., 2016). Dothideomycetes is known to secrete a variety of cellulase and hemicellulose (Wang et al., 2019a,b). Moreover, sugars, soluble starch, and cellulose in litter gradually decomposed as litter decomposition progressed, which lead to an increase in lignin accumulation that results in the colonization of *Aspergillus* and other microbial groups during the latter litter decomposition stage, namely microbial groups that have the capacity to decompose lignin (Bonanomi et al., 2019). *Cladosporium* and *Alternaria* are the dominant fungal genera during the early litter decomposition stage, while *Trichoderma* and *Knufia* significantly increase during the litter decomposition process. Being widely distributed throughout the soil, *Cladosporium* and *Alternaria* can utilize small-sized molecular sugars and starch. *Trichoderma* is a typical cellulolytic fungus with strong cellulose- and hemicellulose-decomposing abilities (Yan, 2011). In this study, *Trichoderma* significantly correlates with heavy metals, sucrase, and catalase, which indicate that this genus possesses a certain level of heavy metal tolerance. Additionally, *Trichoderma* is a highly significant fungal genus in litter decomposition in heavy metal contaminated areas.

### Microbial Interactions During Litter Decomposition

The D100 stage yields the maximum node and edge number values and connectivity values in the fungal network, which is indicative of the complexities of interactions between the fungal communities when reaching their highest values. The nutrient content of litter is higher during the early decomposition stage, and an adequate amount of nutrients promote an increase in microbial biomass, providing greater opportunities for species to interact (Zhou et al., 2020). Results show that resource and information transmission rates are higher, and an increase in functional diversity is higher compared to a relatively simple network (Wagg et al., 2019). Additionally, complex microbial networks also improve fungal tolerance to environmental interference while being able to maintain stable microbial communities (Shang et al., 2018). Overall, litter fungi community stability is highest during the early litter decomposition stage, after which interactions among fungal communities decrease, further confirming that fungal communities may be most active during the prophase stage of litter decomposition.

Furthermore, BC values reflect how nodes connect to other nodes. Generally, the larger the BC value is, the more important role that the node plays in maintaining stable network connectivity (Chen et al., 2019). On a class level, results show that Sordariomycetes and Dothideomycetes gradually replace Leotiomycetes as litter decomposition progressed. This is consistent with the changes observed in fungal community composition. This consistency shows that the interactive ability of more abundant fungi is higher within fungal communities (Zhan et al., 2021). However, it must be noted that the key fungi within our network analysis are based on statistical analysis and therefore do not represent real-world relationships among fungal communities, which remain to be verified (Freilich et al., 2018). Therefore, future studies should selectively exclude some key species in verifying the role they play in species interactions and microbial community functions (Zheng et al., 2021).

### Litter Fungal Community and Litter Factor Relationships

Environmental factors (e.g., litter quality, pH, temperature, and water content) significantly affect the fungal community structure during litter decomposition processes (Chen et al., 2020a, 2021). Results from this study show that the nutrient content, the heavy metal content, and the pH level in litter significantly correlate with fungal community composition. Additionally, the Cu content and the pH level are driving factors of fungal community structure. It has been previously reported that TC and TN content in litter significantly correlates with fungal community structure (Zeng et al., 2017). Moreover, N content contributes to the synthesis of proteins and nucleotides as well as the synthesis of other macromolecules that are vital to physiological microbial functions (Purahong et al., 2015). Thus, N typically acts as a limiting factor in litter decomposition, while also affecting the microbial community structure associated with N transformation processes (Lucas-Borja et al., 2019). In soil ecosystems, pH has been reported to be a key environmental factor that affects microbial community composition and has a considerable effect on microbial community structure and functional pathways (Lucas-Borja et al., 2019). A previous study has shown that the fungal community structure and the functional pathway were both mainly driven by litter pH and TC during litter decomposition (Wang et al., 2019a,b), indicating that pH also plays an important role in litter microbial community assemblages.

Clearly, at low concentrations, heavy metals are necessary for microbial growth, and the role they play in a variety of biological metabolic processes is important (Chen et al., 2020b). However, at higher concentrations, heavy metals impact microbial composition, functional genes, and microbial diversity (Chen et al., 2018). Results from this study show that *Dichotomopilus*, *Trichoderma*, *Knufia*, *Phialophora*, *Oxyporus*, and *Monocillium* significantly and positively correlated with heavy metal, sucrase, and catalase content (*p*<0.05). It can therefore be concluded that soil heavy metal content may affect extracellular enzyme activities by instigating changes in fungal community composition, which will ultimately have an effect on *I. cylindrica* litter decomposition.

## Conclusion

In this study, we found that significant differences were observed in litter decomposition characteristics among the different sub-dams, while the litter decomposition mass was significantly lower in the S536 sub-dam compared to the other sub-dams. *Ascomycetes* was the dominant species in the fungal community during the initial litter decomposition stage. Fungal community diversity significantly decreased as litter decomposition progressed, while degrees of interaction and stability of the fungal communities were highest during the early litter decomposition stage. Finally, significant differences were observed in fungal community structure during the different decomposition stages. The Cu content and the pH value were the key driving factors of fungal community assemblages.

## Data Availability Statement

The datasets presented in this study can be found in online repositories. The names of the repository/repositories and accession number(s) can be found in the article/Supplementary Material.

## Author Contributions

TJ conceived and designed the experiments. TG and XW performed the experiments. BC contributed new reagents. TJ wrote the manuscript. All authors read and approved the manuscript.

## Funding

This study was supported by the National Natural Science Foundation of China (Grant No. 32171524), Shanxi Province Science Foundation for Excellent Young Scholars (Grant No. 201901D211196), Scientific and Technological Innovation Programs of Higher Education Institutions in Shanxi (Grant No.2019 L0005), and Shanxi Province Foundation for Returnees (Grant No. 2021–018), Shanxi Province Graduate Education Innovation Project (Grant No. 2021Y119).

## Conflict of Interest

The authors declare that the research was conducted in the absence of any commercial or financial relationships that could be construed as a potential conflict of interest.

## Publisher’s Note

All claims expressed in this article are solely those of the authors and do not necessarily represent those of their affiliated organizations, or those of the publisher, the editors and the reviewers. Any product that may be evaluated in this article, or claim that may be made by its manufacturer, is not guaranteed or endorsed by the publisher.

## Footnotes

^1^ https://www.ncbi.nlm.nih.gov/sra

^2^ http://drive5.com/usearch/

^3^ http://rdp.cme.msu.edu
