# Supplementary material for: Litter Decomposition of Imperata cylindrica in a Copper Tailing Areas With Different Restoration History: Fungal Community Dynamics and Driving Factors
Source: Front Microbiol. 2021 Nov 22;12:780015. doi: 10.3389/fmicb.2021.780015 (PMC8647173; doi:10.3389/fmicb.2021.780015)
Supplement: Supplementary file 1 [file Data_Sheet_1.PDF]

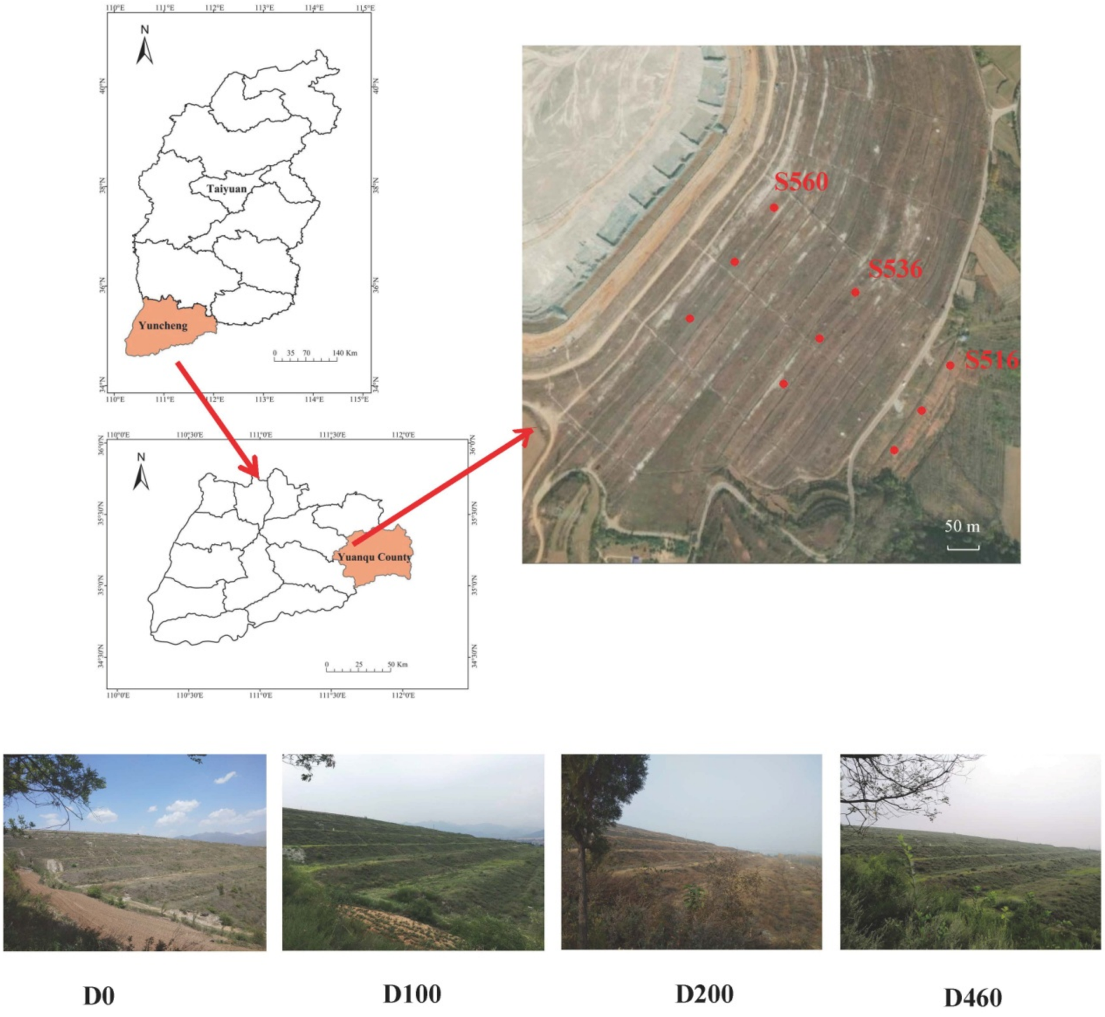

Fig. S1 Sample plots were set up in copper tailings area, and litter samples were collected after being allowed to decompose for 100 days (D100), 200 days (D200), and 460 days (D460).

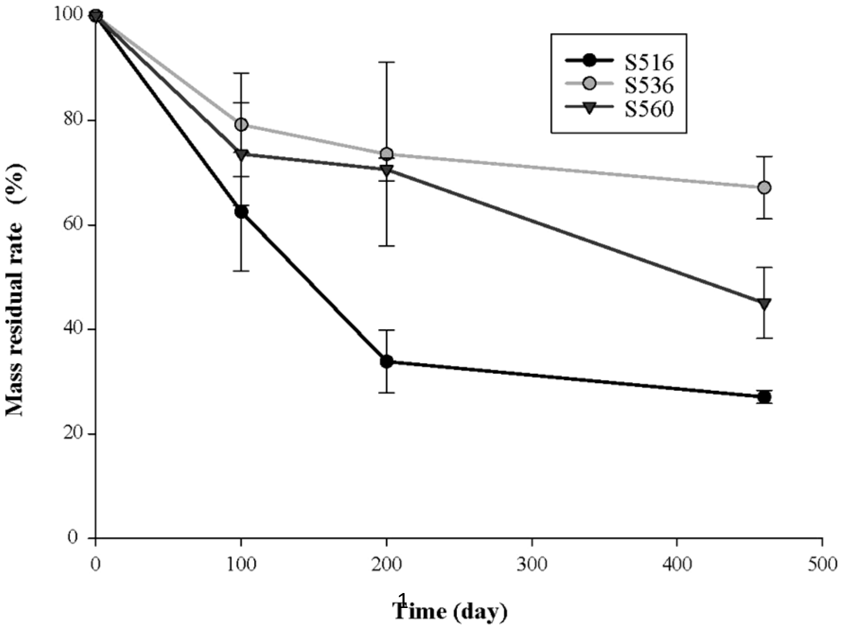

Fig. S2 Mass residual rate of *I. cylindrica* litters from three sub-dams.

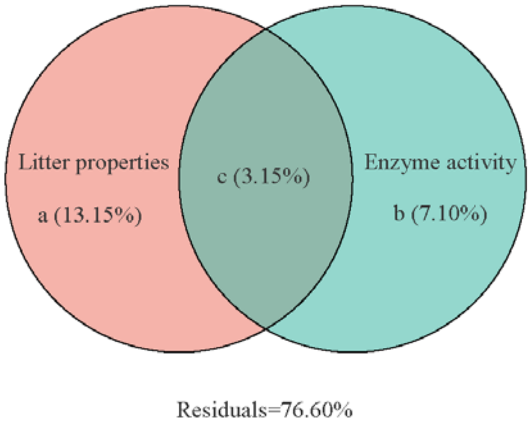

Fig. S3 VPA analysis of litter properties and enzyme activities for differences in litter fungal communities

Table S1 Soil physical and chemical properties of three sub-dams in copper tailings area.

Ecological factor	S516	S536	S560
Soil_TC（%）	2.143 ± 0.788a	0.647 ± 0.193b	0.557 ± 0.207b
Soil_TN（%）	0.143 ± 0.0727a	0.024 ± 0.014b	0.022 ± 0.013b
Soil_C/N	16.346 ± 4.148b	30.198 ± 6.656a	28.382 ± 6.661a
SWC（%）	13.055 ± 7.513a	6.770 ± 2.630ab	5.547 ± 3.138b
Soil_pH	7.944 ± 0.252b	8.114 ± 0.103ab	8.218 ± 0.161a
Soil_Cu（$mg·kg^{-1}$）	418.408 ± 123.080a	347.032 ± 18.937a	487.837 ± 51.097a
Soil_Zn（$mg·kg^{-1}$）	105.606 ± 10.795a	72.359 ± 10.873b	51.276 ± 15.019b
Soil_Pb（$mg·kg^{-1}$）	265.647 ± 31.314a	173.073 ± 37.910a	185.807 ±107.931a
Soil_Cd（$mg·kg^{-1}$）	5.967 ± 0.659b	7.580 ± 0.833a	3.193 ± 0.083c

Data were means ± standard deviations. The different case letters indicated that the means were significantly different among vegetation restoration stages ($P < 0.05$) with Duncan test. Abbreviations: soil water content (SWC), soil total nitrogen (Soil_TN), soil total carbon (Soil_TC) and the ratio of soil carbon nitrogen (Soil_C/N).

Table S2 Properties of litters at the different decomposition stages.

Sub-dam	Decomposition stage	TC %	TN %	C/N	Cu mg·kg^{-1}	Zn mg·kg^{-1}	Pb mg·kg^{-1}	Cd mg·kg^{-1}	pH
S516	D0	43.578 ± 0.083a	1.261 ± 0.052a	34.597 ± 1.470b	11.181 ± 0.290c	26.722 ± 0.323b	1.173 ± 0.158d	0.210 ± 0.034c	6.787 ± 0.006d
	D100	30.771 ± 9.193b	0.463 ± 0.055c	65.482 ± 12.658a	54.480 ± 36.994b	38.150 ± 11.852b	1.565 ± 0.291c	0.249 ± 0.101c	7.077 ± 0.092c
	D200	26.648 ± 8.792c	0.769 ± 0.159b	37.653 ± 21.821b	64.477 ± 7.681b	76.929 ± 11.258a	3.220 ± 0.151b	0.631 ± 0.240b	7.367 ± 0.012b
	D460	16.300 ± 0.191c	0.652 ± 0.014b	25.002 ± 0.227b	175.200 ± 2.221a	65.466 ± 0.468a	16.036 ± 0.134a	1.098 ± 0.147a	7.570 ± 0.010a
S536	D0	43.158 ± 0.113a	0.583 ± 0.097a	75.378 ± 12.721b	13.762 ± 1.225c	18.818 ± 2.727c	1.493 ± 0.276c	0.309 ± 0.027b	6.720 ± 0.010d
	D100	25.407 ± 8.658ab	0.287 ± 0.055b	86.677 ± 14.834a	182.013 ± 77.180b	44.908 ± 13.842ab	1.702 ± 0.128c	0.455 ± 0.200b	6.977 ± 0.006c
	D200	18.382 ± 6.677bc	0.412 ± 0.040b	44.096 ± 14.342b	269.613 ± 94.172ab	64.273 ± 15.119a	3.961 ± 0.366b	0.576 ± 0.137b	7.750 ±0.017a
	D460	12.300 ± 4.594c	0.484 ± 0.174b	25.265 ± 1.193b	317.790 ± 4.654a	43.652 ± 3.590 b	9.810 ± 0.641a	1.042 ± 0.255a	7.663 ± 0.015b
S560	D0	37.722 ± 0.142a	1.188 ± 0.036a	31.761 ± 0.962b	37.097 ± 1.828b	31.299 ± 0.593a	3.095 ± 0.839b	0.443 ± 0.024b	6.417 ±0.006d
	D100	20.231 ±8.310bc	0.506 ± 0.178c	39.255 ± 3.638a	254.773 ± 90.431a	48.535 ± 14.911a	5.572 ± 2.362ab	0.772 ± 0.270a	7.220 ± 0.052c
	D200	15.885 ± 2.999c	0.511 ± 0.068c	30.960 ± 1.659b	309.157 ± 18.502a	54.914 ± 31.363a	9.937 ± 3.963a	0.591 ± 0.074ab	7.717 ± 0.051a
	D460	27.991 ± 2.308b	0.916 ± 0.062b	30.610 ± 2.363b	345.247 ± 56.583a	36.373 ± 0.789a	7.688 ± 1.595ab	0.800±0.129a	7.560 ± 0.017b

Data were means ± standard deviations. The different case letters indicated that the means are significantly different among vegetation restoration stages ($P < 0.05$) with Duncan test.

Table S3 The extracellular enzyme activities of litters during the different decomposition stages.

Sub-dam	Decomposition stage	Urease (mg·(g·24 h)$^{-1}$)	Sucrase (mg·(g·24 h)$^{-1}$)	Cellulase (mg·(g·72 h)$^{-1}$)	Cellulase (mg·(g·20 min)$^{-1}$)
S516	D0	3.369 ± 0.019a	3.310 ± 0.644c	0.811 ± 0.059b	5.626 ± 0.706ab
	D100	0.037 ± 0.005c	9.691 ± 0.383a	0.222 ± 0.025c	3.160 ± 1.766b
	D200	2.966 ± 0.042b	4.769 ± 0.063b	1.101 ± 0.038a	5.086 ± 2.312ab
	D460	3.229 ± 0.112ab	1.737 ± 0.012d	0.221 ± 0.050c	7.321 ± 0.133a
S536	D0	3.760 ± 0.308b	3.072 ± 0.716c	0.772 ± 0.054a	3.237 ± 0.231c
	D100	2.385 ± 0.063d	3.559 ± 0.027c	0.481 ± 0.031c	8.863 ± 1.413a
	D200	4.661 ± 0.064a	4.823 ± 0.119b	0.525 ± 0.026b	5.857 ±1.335b
	D460	2.934 ± 0.042c	6.967 ± 0.040a	0.551 ± 0.023b	5.626 ± 1.355b
S560	D0	2.959 ± 0.331c	2.299 ± 0.330c	0.623 ± 0.033a	0.848 ± 0.133b
	D100	1.160 ± 0.032d	4.216 ± 0.057c	0.383 ± 0.040b	7.707 ± 0.267a
	D200	4.810 ± 0.045a	18.450 ± 0.268a	0.310 ± 0.050c	7.784 ± 0.667a
	D460	4.157 ± 0.027b	12.796 ± 0.051b	0.585 ± 0.021a	8.169 ± 0.812a

Data were means ± standard deviations. The different case letters indicate that the means were significantly different among vegetation restoration stages ($P < 0.05$) with Duncan test.
